# Tick-Borne Disease Infections and Chronic Musculoskeletal Pain

**DOI:** 10.1001/jamanetworkopen.2023.51418

**Published:** 2024-01-11

**Authors:** Diana L. Zychowski, Carolina Alvarez, Haley Abernathy, Dana Giandomenico, Shailesh K. Choudhary, Julia M. Vorobiov, Ross M. Boyce, Amanda E. Nelson, Scott P. Commins

**Affiliations:** 1 Institute for Global Health and Infectious Diseases, University of North Carolina at Chapel Hill, Chapel Hill; 2 Thurston Arthritis Research Center, University of North Carolina at Chapel Hill, Chapel Hill; 3 Department of Epidemiology, Gillings School of Global Public Health, University of North Carolina at Chapel Hill, Chapel Hill; 4 Carolina Population Center, University of North Carolina at Chapel Hill, Chapel Hill; 5 Departments of Medicine and Pediatrics, Division of Allergy and Immunology, University of North Carolina at Chapel Hill, Chapel Hill

## Abstract

**Importance:**

Tick-borne diseases (TBDs) other than Lyme disease, such as spotted fever group rickettsiosis, ehrlichiosis, and galactose-α-1,3-galactose (α-gal) syndrome, are an emerging public health issue. Long-term sequelae secondary to *Ehrlichia* or *Rickettsia* infection are uncommon; however, musculoskeletal symptoms are often attributed to prior tick exposure.

**Objective:**

To evaluate the potential associations between prior exposure to TBDs and musculoskeletal symptoms, including radiographic osteoarthritis.

**Design, Setting, and Participants:**

This cross-sectional study analyzed serum samples from the fourth visit (2017-2018) of the Johnston County Osteoarthritis (JoCo OA) project, an ongoing longitudinal, population-based study in Johnston County, North Carolina. Biospecimen testing and analysis were performed between May 2022 and November 2023. Participants in the JoCo OA project are noninstitutionalized White and Black Johnston County residents 45 years or older.

**Main Outcome and Measures:**

The primary outcome was seropositivity with *Ehrlichia* IgG, *Rickettsia* IgG, and/or α-gal IgE and musculoskeletal symptoms. Secondary outcomes included risk factors associated with elevated α-gal IgE and weighted population point prevalence rates. Participants completed questionnaires, underwent physical assessments, and provided biospecimens for serological testing. Multivariable models were used to estimate associations of interest.

**Results:**

Of the 605 participants who completed the fourth visit of the JoCo OA project, 488 (80.7%) had serum samples available for testing. The 488 participants had a median (IQR) age of 72 (68-78) years and included 336 females (68.9%) and 161 Black (33.0%) and 327 White (67.0%) individuals. The overall weighted point prevalence was 8.6% (95% CI, 5.9%-11.3%) for *Ehrlichia* IgG, 17.1% (95% CI, 12.6%-21.5%) for *Rickettsia* IgG, and 19.6% (95% CI, 15.3%-23.8%) for α-gal IgE level greater than 0.1 IU/mL. Only α-gal IgE was associated with knee pain, aching or stiffness (mean ratio, 1.30; 95% CI, 1.09-1.56). Antibodies to *Rickettsia*, *Ehrlichia*, and α-gal were not associated with symptomatic radiographic knee osteoarthritis. Male sex (odds ratio [OR], 2.63; 95% CI, 1.55-4.47), current smoker status (OR, 3.55; 95% CI, 1.38-9.18), and an attached tick bite in the past 5 years (OR, 3.99; 95% CI, 2.22-7.15) were all risk factors that were associated with α-gal IgE level greater than 0.1 IU/mL. Despite only 84 individuals (17.2%) recalling a tick bite in the past 5 years, 178 (36.5%) had evidence of prior tick-borne exposure, suggesting frequent human-tick interactions.

**Conclusions and Relevance:**

Results of this cross-sectional study indicate no association between *Ehrlichia* or *Rickettsia* seropositivity and chronic musculoskeletal symptoms or osteoarthritis. Further investigation is needed into the pathogenesis of α-gal syndrome and interventions to reduce human-tick interactions.

## Key Points

**Question:**

What is the seroprevalence of *Rickettsia*, *Ehrlichia,* and Galactose-α-1,3-galactose (α-gal) antibodies, and is exposure to these tick-borne diseases (TBDs) associated with chronic musculoskeletal symptoms?

**Findings:**

In this cross-sectional study of 488 individuals, the weighted point prevalence was approximately 9% for *Ehrlichia* IgG, 17% for *Rickettsia* IgG, and 20% for α-gal IgE. Antibodies to these TBDs were not associated with symptomatic radiographic knee osteoarthritis, but individuals with elevated α-gal IgE levels reported higher rates of knee pain, aching, or stiffness.

**Meaning:**

Findings from this study suggest that *Ehrlichia* or *Rickettsia* seropositivity was not associated with musculoskeletal symptoms but further investigation into the pathogenesis of α-gal syndrome is needed.

## Teaser

This cross-sectional study examines the prevalence of spotted fever group rickettsiosis, ehrlichiosis, and α-gal syndrome and their association with symptomatic radiographic knee osteoarthritis in adults 45 years or older.

## Introduction

More than 50 000 cases of tick-borne disease (TBD) were reported in the US in 2019, representing a doubling of cases over the prior 2 decades.^1,2^ Yet surveillance systems almost certainly underestimate the true incidence of infection.^3^ While Lyme disease accounts for the majority of reported TBD cases, spotted fever group rickettsiosis and ehrlichiosis remain important public health issues, particularly in southeastern states. For example, cases of ehrlichiosis, transmitted by the lone star tick (*Amblyomma americanum*), have increased more than 10-fold since 2000.^4^ As ticks continue to expand their geographic range, the incidence of TBD will likely continue to increase.^5,6,7^

Clinical manifestations of spotted fever group rickettsiosis and ehrlichiosis include fever, rash or eschar, headache, myalgia, and arthralgia.^8^ Rocky Mountain spotted fever (RMSF), caused by infection with *Rickettsia rickettsii*, is historically considered to have the highest fatality rate,^9^ but more than half of ehrlichiosis cases require hospitalization, with case fatality rates that can exceed 10% in immunocompromised populations.^10^ Severe RMSF can have long-term neurological sequelae believed to be associated with vascular damage in the central nervous system.^11^ Yet there is no evidence that spotted fever group rickettsiosis or ehrlichiosis establish chronic infections and that, when treated early and appropriately, the vast majority of patients recover fully.^12^

Despite the lack of evidence that infection with *Rickettsia* or *Ehrlichia* is associated with persistent symptoms after treatment, many patients attribute nonspecific symptoms, especially musculoskeletal symptoms, to a prior infection. Several factors explain this discrepancy between current medical knowledge and lay community perceptions. The leading factor is the potential spillover from Lyme disease, the most common tick-borne infection. Lyme arthritis is a well-described phenomenon, even though it typically presents as monoarticular disease rather than diffuse polyarthralgia. Additionally, the substantial media coverage of posttreatment Lyme disease, often referred to as chronic Lyme, likely alters the assumptions regarding other TBDs.

However, there may be a physiological basis for the association between tick bites and arthralgia. In addition to tick-borne infections, lone star tick bites are also associated with the development of IgE to galactose-α-1,3-galactose (α-gal). In some individuals, α-gal IgE results in a delayed-onset reaction after ingestion of mammalian meat or its products, known as α-gal syndrome (AGS).^13,14^ Awareness of and testing for AGS appear to be increasing, with more than 90 000 individuals receiving positive test results between 2017 and 2022.^15^ The most overt symptoms are similar to a typical food allergy, including angioedema, urticaria, and anaphylaxis.^16^ In contrast, some individuals with elevated α-gal IgE levels may not experience any symptoms and are categorized as sensitized to α-gal. There are also more subtle manifestations, including persistent induration and erythema around tick bites; gastrointestinal distress; and arthralgia, which can be diffuse, migratory, and improved by an appropriate avoidance diet.^17,18^

Therefore, the goal of this study was to evaluate the potential associations between prior exposure to TBDs endemic to the southeastern US, including AGS, and chronic musculoskeletal symptoms and radiographic measures of osteoarthritis. To accomplish this, we measured the seroprevalence of *Rickettsia*- and *Ehrlichia*-specific IgG as well as α-gal IgE in a longitudinal, population-based cohort of adults in central North Carolina. We hypothesized that there would be (1) relatively high levels of concurrent seropositivity among individuals with *Ehrlichia* IgG and α-gal IgE given the shared vector, (2) evidence of no association between prior *Rickettsia* and/or *Ehrlichia* exposure and reported joint pain or osteoarthritis, and (3) self-reported arthralgias from individuals with detectable α-gal IgE levels.

## Methods

### Study Site and Design

This cross-sectional study analyzed serum samples from the fourth visit of the Johnston County Osteoarthritis (JoCo OA) project. The JoCo OA project is an ongoing longitudinal, population-based study involving noninstitutionalized White and Black residents 45 years or older from Johnston County, North Carolina. Johnston County is composed of mixed urban and rural areas located in central North Carolina,^19^ an area known to be endemic with TBDs.^1^ The University of North Carolina at Chapel Hill Institutional Review Board approved this study. Both oral and written informed consent were obtained from participants. We followed the Strengthening the Reporting of Observational Studies in Epidemiology (STROBE) reporting guideline.

Details regarding the sampling strategy and recruitment methods are described elsewhere.^20^ In brief, JoCo OA participants self-report demographic characteristics, symptoms, and health status in questionnaires; undergo a physical examination and provide blood samples; and complete performance-based assessments. The serum samples in the present study were obtained from January 2017 to December 2018. Biospecimen testing and analysis were performed between May 2022 and November 2023.

### Laboratory Procedures

Serum samples were screened for IgG antibodies specific to *Ehrlichia chaffeensis* at a 1:64 dilution using an indirect immunofluorescence assay performed according to the manufacturer’s instructions (Fuller Laboratories). Reactive samples were tested again at the following dilutions to ascertain the degree of positivity: 1:64, 1:128, 1:256, 1:512, and 1:1024.

Serum samples were screened for IgG antibodies specific to spotted fever group *Rickettsia* (SFGR) at a 1:100 dilution using an enzyme immunoassay performed according to the manufacturer’s instructions (Fuller Laboratories). Samples with positive or indeterminate test results underwent confirmatory testing via immunofluorescence assay at the University of North Carolina Hospitals McLendon Laboratory using the following dilutions: 1:64, 1:128, 1:256, 1:512, and 1:1024.

Total IgE and serum α-gal IgE antibodies were measured using commercially available ImmunoCAP assays performed with the ImmunoCAP 250 instrument (Phadia 250; Thermo Fisher Scientific) and expressed as international units per milliliter (IU/mL). Serum samples were assayed for IgE to α-gal (o215) per manufacturer instructions, and the cutoff for a positive test result was the limit of detection: greater than 0.1 IU/mL. Response units less than 0.1 IU/mL were noted and extrapolated to form the basis for reporting below the clinical assay threshold.

### Musculoskeletal Symptoms and Osteoarthritis Measures

Self-reported joint symptoms were defined by the question, “On most days of any 1 month in the last 12 months did you have pain, aching, or stiffness in your right/left joint?” Knees, hands, and hips were analyzed. Participants self-reported their pain, aching, or stiffness (PAS) severity on a scale of 0 (none) to 10. The sum of a person’s right and left joint scores defined the PAS severity count, ranging from 0 to 20. The Knee Injury and Osteoarthritis Outcome Score (KOOS) includes the 5 subscales of symptoms, stiffness, pain, physical function, and quality of life and is scored from 0 to 100, with 0 representing extreme problems and 100 representing no problems.^21^ Standardized fixed-flexion posteroanterior knee radiographic images were read by 1 expert musculoskeletal radiologist. Radiographic knee osteoarthritis (RKOA) was defined by the Kellgren-Lawrence grade of 0 to 4, with 0 representing definite radiographic absence of osteoarthritis^22^ and 2 or higher being used to indicate RKOA. Symptomatic RKOA was defined when both symptoms and RKOA were present in the same joint.

### Statistical Analysis

Descriptive statistics were computed, with counts and percentages provided for categorical variables, means and SDs provided for continuous variables, and medians and IQRs provided for count or score variables. Categorical associations between variables were tested using the χ^2^ test for expected cell frequencies higher than 5 and Fisher exact test for cell frequencies lower than 5. Sampling weights were calculated as previously described^23^ to ascertain overall weighted seroprevalence estimates (reflective of population prevalence) and were stratified by age group, sex, race and ethnicity, and geographic region.

The dichotomous outcome of α-gal IgE level greater than 0.1 IU/mL was modeled using logistic regression to produce adjusted odds ratios (ORs) and 95% CIs. Multivariable models were used to assess the independent association of TBD seroprevalence with hand, hip, and knee joint PAS severity count, symptomatic RKOA, and KOOS subscale scores. These models were adjusted for age, sex, race and ethnicity, body mass index, educational level, smoking status, alcohol use, and Charlson Comorbidity Index (score range: 0 to ≥3, with higher scores indicating increased risk of morbidity from comorbid diseases). Further details on models are provided in the eMethods in Supplement 1. Sociodemographic information, such as age, race and ethnicity, and educational level, was self-reported. Due to differences in osteoarthritis incidence rates for certain groups, these variables were included in this study.

Two-sided *P* < .05 indicated statistical significance. Data were analyzed with SAS, version 9.4 (SAS Institute Inc).

## Results

A total of 605 participants completed the fourth visit of the JoCo OA project, of whom 488 (80.7%) had sufficient serum samples for testing (Table 1). These participants had a median (IQR) age of 72 (68-78) years and included 336 females (68.9%) and 152 males (31.1%) as well as 161 Black (33.0%) and 327 White (67.0%) individuals. Three hundred seventy-four participants (76.6%) described their employment status as retired. Regarding osteoarthritis symptoms, 168 participants (34.4%) reported PAS in their hands, 150 participants (30.7%) reported PAS in their hips, and 199 participants (40.8%) reported PAS in their knees in the preceding 12 months (Table 2).

**Table 1.** Baseline Demographic and Clinical Characteristics of Study Participants

Characteristic	Participants, No. (%)^a^
Participants with serum samples, No.	488
Age, median (IQR), y	72 (68-78)
Sex	
Male	152 (31.1)
Female	336 (68.9)
Race and ethnicity^b^	
Black	161 (33.0)
White	327 (67.0)
Educational level	
<High school diploma	59 (12.1)
≥High school diploma	427 (87.5)
Missing data	2 (0.4)
Employment status	
Employed	73 (15.0)
Retired	374 (76.6)
Disabled	31 (6.4)
Other^c^	10 (2.0)
Marital status	
Divorced	60 (12.3)
Married	270 (55.3)
Never married	24 (4.9)
Separated	6 (1.2)
Widowed	128 (26.2)
Annual family income, US$	
<45 000	251 (51.4)
≥45 000	164 (33.6)
Missing data	73 (15.0)
Alcohol use	
Current drinker	88 (18.0)
Nondrinker	399 (81.8)
Missing data	1 (0.2)
Smoking status	
Current smoker	28 (5.7)
Nonsmoker	460 (94.3)
BMI	
Underweight: <18.5	1 (0.2)
Healthy weight: 18.5-24.9	85 (17.4)
Overweight: 25.0-29.9	150 (30.7)
Obese: ≥30.0	252 (51.6)
CCI	
0	197 (40.4)
1	149 (30.5)
2	63 (12.9)
≥3	79 (16.2)
Age-adjusted CCI	
0	0
1	7 (1.4)
2	75 (15.4)
≥3	406 (83.2)
Blood pressure	
Elevated^d^	167 (34.2)
Normal	314 (64.3)
Unknown	7 (1.4)
Diabetes status	
With diabetes	153 (31.4)
Without diabetes	333 (68.2)
Unknown	2 (0.4)
CKD status	
With CKD	16 (3.3)
Without CKD	460 (94.3)
Unknown	12 (2.5)

Abbreviations: BMI, body mass index (calculated as weight in kilograms divided by height in meters squared); CCI, Charlson Comorbidity Index (score range: 0 to ≥3, with higher scores indicating increased risk of morbidity from comorbid diseases); CKD, chronic kidney disease.

^a^ Percentages were rounded and may not total to 100.

^b^ Race and ethnicity were self-reported in the Johnston County Osteoarthritis project.

^c^ Other employment status includes never worked outside the home, do not know, and refused to answer.

^d^ Elevated blood pressure was defined as systolic blood pressure of 140 mm Hg or higher and/or diastolic blood pressure of 90 mm Hg or higher.

**Table 2.** Participant Survey Questions Regarding Tick-Borne Exposure and Musculoskeletal Symptoms

Variable	Participants, No. (%) (n = 488)
Have you had an attached tick bite in the last 5 y?	
Yes	84 (17.2)
No	403 (82.6)
Missing data or NA	1 (0.2)
For those with an attached tick bite in the last 5 y, was there redness, itching or inflammation at the site of the bite?	
Yes	59 of 84 (70.2)
No	25 of 84 (29.8)
For those with an attached tick bite in the last 5 y who experienced redness, itching or inflammation at the site of the bite, did the reaction last less than a week?	
Yes	38 of 59 (64.4)
No	21 of 59 (35.6)
Do you think you have a food allergy?	
Yes	50 (10.2)
No	437 (89.5)
Missing data or NA	1 (0.2)
Are there any foods you avoid because of a physical reaction?	
Yes	70 (14.3)
No	417 (85.5)
Missing data or NA	1 (0.2)
Do you break out in hives sometimes?	
Yes	40 (8.2)
No	447 (91.6)
Missing data or NA	1 (0.2)
Do you have a reaction to tick bites?	
Yes	8 (1.6)
No	288 (59.0)
Missing data or NA	192 (39.3)
Do you have a reaction to insect bites or spider bites?	
Yes	44 (9.0)
No	400 (82.0)
Missing data or NA	44 (9.0)
Do you have a reaction to stings?	
Yes	107 (21.9)
No	347 (71.1)
Missing data or NA	34 (7.0)
Do you eat red meat (this includes beef, pork, venison, lamb)?	
Yes	464 (95.1)
No	23 (4.7)
Missing data or NA	1 (0.2)
Have you been diagnosed with [α-gal] allergy to red meat?	
Yes	12 (2.5)
No	475 (97.3)
Missing data or NA	1 (0.2)
Have you had hand PAS in the past year?	
Yes	168 (34.4)
No	320 (65.6)
Hands PAS severity count, median (IQR)	0 (0-6)
Have you had hip PAS in the past year?	
Yes	150 (30.7)
No	338 (69.3)
Hips PAS severity count, median (IQR)	0 (0-5)
Have you had knee PAS in the past year?	
Yes	199 (40.8)
No	289 (59.2)
Knee PAS severity count, median (IQR)	0 (0-7)

Abbreviations: α-gal, galactose-α-1,3-galactose; NA, not applicable; PAS, pain, aching, or stiffness.

### TBD Exposure

Of the individuals tested, 84 (17.2%) recalled a tick bite in the past 5 years and 59 (70.2%) of these 84 individuals experienced redness, itching, or inflammation at the attachment site (Table 2). In total, 77 of 488 participants (15.8%) had detectable IgG antibodies to SFGR. Reciprocal titer levels ranged from 1:64 to 1:512, with 1:64 (38 [49.4%]) and 1:128 (24 [31.2%]) being the most common. Only 15 of 77 participants (19.5%) had titer levels of 1:256 or higher. Forty-five of 488 participants (9.2%) had detectable IgG antibodies to *Ehrlichia*, although a higher proportion (37.8% [17 of 45]) had titer levels at 1:256 or higher (eFigure 1 in Supplement 1).

A total of 197 participants (40.4%) had detectable IgE to α-gal (>0 IU/mL), whereas 94 participants (19.3%) had a clinically relevant α-gal, defined as IgE level greater than 0.1 IU/mL (eTable 1 in Supplement 1). However, only 12 of 488 individuals (2.5%) reported a diagnosis history of α-gal allergy (Table 2), and 10 of the 12 had α-gal IgE level less than 0.1 IU/mL. Of the 21 individuals with α-gal IgE levels greater than 2 IU/mL, only 1 reported a diagnosis of AGS. Male sex (OR, 2.63; 95% CI, 1.55-4.47), current smoker status (OR, 3.55; 95% CI, 1.38-9.18), and an attached tick bite in the past 5 years (OR, 3.99; 95% CI, 2.22-7.15) were all risk factors that were statistically associated with α-gal IgE level greater than 0.1 IU/mL. However, when stratified by sex, current smoker status and an attached tick bite in the past 5 years were significant for females only (Table 3). Of the 94 individuals with α-gal IgE level greater than 0.1 IU/mL, 7 (7.4%) also had detectable *Ehrlichia* IgG, and 28 (29.8%) had detectable IgG antibodies to SFGR. Most participants (60 of 94 [63.8%]) with α-gal IgE level greater than 0.1 IU/mL did not have evidence of prior infection with either SFGR or *Ehrlichia* (eFigure 2 in Supplement 1). Overall, 178 of 488 individuals (36.5%) had exposure to at least 1 TBD.

**Table 3.** Multivariable Logistic Regression of Association Between Demographic, Clinical, and Serologic Tick-Borne Variables and α-Gal IgE Level Greater Than 0.1 IU/mL

Covariable	OR (95% CI)		
	Overall	Females	Males
Age, 1 y older	1.03 (0.99-1.06)	1.07 (1.01-1.12)	0.99 (0.93-1.05)
BMI, 1 higher	1.00 (0.96-1.05)	1.01 (0.95-1.08)	1.02 (0.96-1.09)
Male sex	2.63 (1.55-4.47)	NA	NA
Black race and ethnicity	0.70 (0.37-1.33)	0.38 (0.15-1.00)	1.12 (0.43-2.92)
<High school educational level	1.22 (0.55-2.69)	0.85 (0.24-3.05)	1.73 (0.55-5.40)
Current smoker	3.55 (1.38-9.18)	6.07 (1.47-25.0)	1.70 (0.44-6.63)
Current alcohol drinker	1.44 (0.77-2.70)	0.87 (0.30-2.56)	2.15 (0.90-5.13)
CCI, 1 unit higher	0.99 (0.84-1.16)	0.96 (0.72-1.28)	0.99 (0.79-1.24)
Has had attached tick bite in past 5 y	3.99 (2.22-7.15)	7.01 (3.08-16.0)	2.37 (0.95-5.90)
Has food allergy	1.20 (0.47-3.06)	2.51 (0.53-12.0)	Not estimable
Avoids foods because of physical reaction	0.88 (0.36-2.12)	0.52 (0.13-2.19)	Not estimable
Breaks out in hives sometimes	1.69 (0.68-4.21)	3.10 (1.01-9.53)	Not estimable
Uses antacid	0.82 (0.47-1.42)	0.95 (0.42-2.14)	0.82 (0.36-1.85)
Has heartburn or pain related to eating	1.22 (0.67-2.21)	0.98 (0.42-2.29)	1.50 (0.57-3.96)
Has reaction to tick bites, insect or spider bites, or stings	1.12 (0.60-2.07)	1.06 (0.44-2.56)	1.50 (0.57-3.94)
Eats red meat >1 time per wk	1.33 (0.78-2.28)	1.92 (0.87-4.24)	0.99 (0.42-2.34)
Has α-gal allergy to red meat	0.59 (0.11-3.17)	Not estimable	Not estimable
Positive *Ehrlichia* IgG test result	0.91 (0.36-2.34)	Not estimable	Not estimable
Positive SFGR IgG test result	1.96 (1.07-3.60)	0.95 (0.30-2.99)	2.63 (1.17-5.92)

Abbreviations: α-gal, galactose-α-1,3-galactose; BMI, body mass index; CCI, Charlson Comorbidity Index; NA, not applicable; OR, odds ratio; SFGR, spotted fever group *Rickettsia*.

Compared with females, males were substantially more likely to have detectable antibodies for SFGR (OR, 3.28; 95% CI, 1.99-5.41) and α-gal IgE level greater than 0.1 IU/mL (OR, 3.25; 95% CI, 2.05-5.17). Those with an attached tick bite in the past 5 years had a higher likelihood of detectable SFGR antibodies (OR, 2.44; 95% CI, 1.39-4.26). No associations were observed between antibodies to *Ehrlichia*, SFGR, and α-gal IgE level greater than 0.1 IU/mL and baseline comorbidities, including age-adjusted Charlson Comorbidity Index (eTable 2 in Supplement 1).

When modeling for population exposures in Johnston County, North Carolina, the weighted point prevalence among adults 45 years or older was 8.6% (95% CI, 5.9%-11.3%) for *Ehrlichia*, 17.1% (95% CI, 12.6%-21.5%) for SFGR, and 19.6% (95% CI, 15.3%-23.8%) for an α-gal IgE level greater than 0.1 IU/mL (Table 4).

**Table 4.** Weighted Point Prevalence Estimates for Positive Spotted Fever Group *Rickettsia* (SFGR) IgG Test Result, Positive *Ehrlichia* IgG Test Result, and α-Gal IgE Level Greater Than 0.1 IU/mL

Strata	Unweighted Count	Weighted point prevalence estimates (95% CI), %		
		Positive *Ehrlichia* IgG test result	Positive SFGR IgG test result	α-Gal IgE level >0.1 IU/mL
All participants^a^	488	8.6 (5.9-11.3)	17.1 (12.6-21.5)	19.6 (15.3-23.8)
Age group, y				
55-64	54	12.6 (3.9-21.2)	12.8 (3.4-22.2)	14.5 (5.2-23.9)
65-74	249	5.6 (2.9-8.3)	18.8 (12.0-25.5)	21.0 (14.3-27.7)
75-84	145	12.3 (6.1-18.5)	14.3 (7.8-20.8)	17.2 (10.4-24.0)
≥85	40	5.7 (0.0-12.6)	26.7 (6.8-46.6)	33.4 (13.6-53.1)
Sex	336	10.7 (6.7-14.7)	9.0 (4.8-13.3)	12.6 (8.3-16.8)
Female				
Male	152	5.0 (2.2-7.9)	30.4 (21.4-39.4)	31.2 (22.5-40.0)
Race and ethnicity				
Black	159	12.9 (6.6-19.3)	13.6 (7.1-20.1)	15.2 (8.7-21.7)
White	329	7.5 (4.4-10.6)	17.9 (12.7-23.1)	20.7 (15.6-25.8)
Geographic region				
Urban	152	9.1 (4.6-13.6)	17.0 (10.0-24.1)	14.6 (7.6-21.6)
Rural	72	8.5 (3.5-13.6)	22.2 (10.1-34.4)	24.0 (11.6-36.5)

Abbreviation: α-gal, galactose-α-1,3-galactose.

^a^ Data were weighted to the 2010 US Census data for Johnston County, North Carolina.

### Associations Between TBD and Musculoskeletal Symptoms

Multivariable models demonstrated that α-gal IgE level greater than 0.1 IU/mL was associated with an increased mean ratio (MR) for knees PAS (MR, 1.30; 95% CI, 1.09-1.56), but similar associations were not observed with the hands or hips PAS. There were no associations with a detectable *Ehrlichia* or SFGR IgG and PAS at any joint.

Detectable antibodies to *Ehrlichia*, SFGR, or α-gal IgE level greater than 0.1 IU/mL were not statistically associated with symptomatic RKOA (Table 5). While α-gal IgE level greater than 0.1 IU/mL was associated with increased KOOS for pain (MR, 1.31; 95% CI, 1.05-1.62) and quality of life (MR, 1.18; 95% CI, 1.03-1.35), there were no associations between antibodies to *Ehrlichia* or SFGR and increased KOOS for pain and quality of life (eTable 3 in Supplement 1). As an exploratory analysis, we stratified by antibody level and found that only α-gal IgE level greater than 0.35 IU/mL was significant for knees PAS severity (MR, 1.49; 95% CI, 1.19-1.85) (eTable 4 in Supplement 1).

**Table 5.** Multivariable Models for Pain, Aching, or Stiffness (PAS) and Symptomatic Radiographic Knee Osteoarthritis (RKOA)

Covariable^a^	PAS, MR (95% CI)			Symptomatic RKOA, OR (95% CI)
	Hands	Hips	Knees	
Positive *Ehrlichia* IgG test result	0.88 (0.65-1.17)	0.84 (0.66-1.07)	0.98 (0.73-1.32)	0.69 (0.30-1.60)
Positive SFGR IgG test result	1.14 (0.95-1.37)	0.89 (0.72-1.11)	1.05 (0.88-1.26)	1.65 (0.92-2.98)
α-Gal IgE level >0.1 IU/mL	1.19 (1.00-1.42)	1.18 (0.98-1.43)	1.30 (1.09-1.56)	0.78 (0.43-1.43)

Abbreviations: α-gal, galactose-α-1,3-galactose; MR, mean ratio; OR, odds ratio; SFGR, spotted fever group *Rickettsia*.

^a^ Models adjusted for age, body mass index, sex, race and ethnicity, educational level, smoking status, alcohol use, Charlson Comorbidity Index, and other listed covariates.

## Discussion

To our knowledge, this study was the first population-based seroprevalence study of SFGR, *Ehrlichia*, and α-gal. The study revealed a high prevalence of TBD exposure among adult JoCo OA project participants in central North Carolina. Despite only 84 individuals (17.2%) recalling a tick bite in the past 5 years, 178 (36.5%) had either α-gal IgE level greater than 0.1 IU/mL, a positive test result for SFGR IgG antibodies, or a positive test result for *Ehrlichia* IgG antibodies, suggesting high levels of tick exposure in this cohort. Given that not every tick is a vector of an infectious pathogen or an allergen (for example, entomological studies suggest that <10% of lone star ticks are infected with *Ehrlichia*^24^), the findings highlight that human-tick interactions are common.

While no dose-response association was seen for *Ehrlichia* or SFGR antibodies and any joint PAS, higher α-gal IgE levels were associated with knees PAS severity (MR, 1.49; 95% CI, 1.19-1.85). Compared with titer level lower than 1:64, SFGR IgG at 1:64 was associated with symptomatic RKOA. This association was likely spurious and secondary to small counts and unaccounted covariates, as it was not redemonstrated for SFGR higher than 1:64.

Overall, we did not find associations between prior infection with SFGR or *Ehrlichia* exposure and musculoskeletal symptoms or radiographic markers of osteoarthritis. These results are reassuring and provide valuable evidence to guide patients. The association between knee pain and α-gal IgE, however, is intriguing and merits further investigation.

Acknowledging differences in study design, the results suggest that adult residents of Johnston County, North Carolina, had similar or higher seroprevalence of antibodies against *Ehrlichia* and SFGR compared with previous findings in other endemic areas or among individuals with high-risk occupational exposures.^25,26,27,28,29^ This difference may be partly attributable to the older age of participants in the JoCo OA project, which has been associated with higher seroprevalence, likely due to greater years at risk of exposure. We initially hypothesized that there would be substantial overlap between *Ehrlichia* and α-gal exposures, as both are primarily transmitted by the same tick vector. However, as shown in eFigure 1 in Supplement 1, relatively few individuals (7 of 488 [1.4%]) had detectable *Ehrlichia* IgG and α-gal IgE level greater than 0.1 IU/mL. There was a greater overlap between positive test results for α-gal and SFGR antibodies. Additionally, as shown in eFigure 1 in Supplement 1, 36% of participants (28 of 77) with detectable SFGR IgG had α-gal IgE level greater than 0.1 IU/mL. Given that the lone star tick is believed to be a poor vector of *R rickettsii,* the infectious agent responsible for RMSF,^12,30^ it is likely that the high prevalence of SFGR antibodies is associated with cross-reactivity with other SFGRs, such as *Rickettsia amblyommatis,* which is also found in lone star ticks.^31,32^ In North Carolina, *R. amblyommatis* is the most common rickettsial exposure, accounting for more than 80% of the *Rickettsia* species found in ticks with positive test result for 17-kDa protein.^24^ While the clinical outcome of *R amblyommatis* infection remains poorly described,^33,34^ exposure is likely followed by seroconversion, which would explain both the high prevalence in the study population and the overlap with α-gal IgE serum levels.

To our knowledge, this study is the largest population-based serosurveillance study of α-gal in the US to date. Male sex, female sex with current smoking status, and female sex with an attached tick bite in the past 5 years were all factors independently associated with increased odds of α-gal IgE level greater than 0.1 IU/mL. It is uncertain whether the association between current smoking status in females and α-gal IgE level greater than 0.1 IU/mL represents an unknown biological mechanism or is a proxy for outdoor exposure. Further evaluation in larger cohorts is warranted.

We found a weighted prevalence of 19.6% (95% CI, 15.3%-23.8%) for α-gal IgE level greater than 0.1 IU/mL, similar to other cohorts where prevalence ranged from 12% to 31%.^35,36,37,38,39^ The prevalence was even higher among males (31.2%; 95% CI, 22.5%-40.0%), although it is uncertain whether this difference was primarily biological or behavioral (eg, high-risk outdoor activity). The study population uniquely differed from participants in all prior α-gal studies in that we tested serum samples derived from a population-based cohort and did not specifically select individuals with risk factors associated with elevated α-gal IgE levels.

In this cohort, 197 (40.4%) of screened individuals had detectable IgE antibodies to α-gal (eg, >0.0 IU/mL), whereas 94 (19.3%) had α-gal IgE level greater than 0.1 IU/mL. Since the laboratory threshold to detect α-gal IgE is routinely set to 0.1 IU/mL, there were no available data on the clinical implications, if any, of low α-gal IgE levels below 0.1 IU/mL. Those with α-gal IgE above 0 IU/mL but below 0.1 IU/mL may be immunologically sensitized even as the phenotypic manifestations of such a finding are unknown. However, sensitization to α-gal, even at elevated serum levels, may not reflect symptomatic disease. For example, only 8.6% of German forest service employees and hunters with detectable α-gal IgE level of 0.35 IU/mL or greater experienced allergic reactions to mammalian meat or its derivatives.^40^ Furthermore, even in individuals with a clinical diagnosis of AGS, the level of α-gal IgE was not directly associated with disease severity but may decrease over time for those who avoid recurrent tick bites.^16,37^ In the present cohort, 12 of 488 individuals (2.5%) self-reported a diagnosis of AGS, and 10 of the 12 had an α-gal IgE level less than 0.1 IU/mL. It is possible that these 10 individuals had decreased α-gal serum levels due to tick avoidance. On the other hand, only 1 of 21 individuals (4.8%) with α-gal IgE level greater than 2 IU/mL self-reported an AGS diagnosis. These data highlight the ubiquitous sensitization to α-gal within certain locales, the likely underdiagnosis of AGS within this population, and the opportunities that clinicians have to educate patients on tick bite prevention.

### Strengths and Limitations

This study has strengths. It had the ability to leverage an established longitudinal cohort with a complex sampling strategy that provided weighted population prevalence rates for *Ehrlichia,* SFGR, and α-gal exposure. We are not aware of a comparable cohort wherein participants are enrolled on the basis of place of residence within a similar area of high exposure to multiple tick species.

The study and findings are subject to limitations, however. First, a single, reactive IgG titer does not equate to clinical disease. The study was not designed to capture a history of typical symptoms or prior clinical diagnoses. Therefore, it is not appropriate to generalize the findings to estimate disease burden. The results are further complicated by cross-reactivity between different *Ehrlichia* or SFGR species. Second, because IgG may remain elevated for years after exposure, we were not able to establish temporal associations between exposure and musculoskeletal symptoms. However, we are reassured by the absence of any association between musculoskeletal symptoms and tick-borne infections.

## Conclusions

In this cross-sectional study of a population with high levels of tick exposure and osteoarthritis, there was no association between musculoskeletal symptoms and SFGR and *Ehrlichia* antibodies. However, α-gal IgE was associated with knee pain, aching, or stiffness. These findings suggest that substantial investment is required to examine the pathogenesis of these TBDs and interventions to reduce human-tick interactions.

## Notes

Supplement 1. **eMethods.** Details on Multivariable Models **eFigure 1.** Ehrlichia and SFGR Testing Flowsheet **eFigure 2.** Distribution of Tick-Borne Serologic Data **eTable 1.** Alpha-Gal and Total IgE Levels **eTable 2.** Univariate Analyses **eTable 3.** Multivariable Models for KOOS **eTable 4.** Multivariable Models for Symptomatic Radiographic Osteoarthritis As Well As Hands, Hips and Knees PAS

Supplement 2.
Data Sharing Statement
