# Supplementary material for: Tick-Borne Disease Infections and Chronic Musculoskeletal Pain
Source: JAMA Netw Open. 2024 Jan 11;7(1):e2351418. doi: 10.1001/jamanetworkopen.2023.51418 (PMC10784854; doi:10.1001/jamanetworkopen.2023.51418)
Supplement: Supplement 1. — eMethods. Details on Multivariable Models eFigure 1. Ehrlichia and SFGR Testing Flowsheet eFigure 2. Distribution of Tick-Borne Serologic Data eTable 1. Alpha-Gal and Total IgE Levels eTable 2. Univariate Analyses eTable 3. Multivariable Models for KOOS eTable 4. Multivariable Models for Symptomatic Radiographic Osteoarthritis As Well As Hands, Hips and Knees PAS [file jamanetwopen-e2351418-s001.pdf]

Supplemental Online Content

Zychowski DL, Alvarez C, Abernathy H, et al. Tick-borne disease infections and chronic musculoskeletal pain. *JAMA Netw Open*. 2024;7(1):e2351418.
doi:10.1001/jamanetworkopen.2023.51418

eMethods. Details on Multivariable Models

eFigure 1. Ehrlichia and SFGR Testing Flowsheet

eFigure 2. Distribution of Tick-Borne Serologic Data

eTable 1. Alpha-Gal and Total IgE Levels

eTable 2. Univariate Analyses

eTable 3. Multivariable Models for KOOS

eTable 4. Multivariable Models for Symptomatic Radiographic Osteoarthritis As Well As Hands, Hips and Knees PAS

This supplemental material has been provided by the authors to give readers additional information about their work.

eMethods. Details on multivariable models

Multivariable models were used to assess the independent association of TBD seroprevalence with hand, hip, and knee joint PAS severity count and symptomatic rKOA. These models were adjusted for age, sex, race, BMI, education, smoking, alcohol use, Charlson comorbidity index. The dichotomous outcome of symptomatic rKOA was modeled using logistic regression to produce adjusted OR and 95% CI for positive *Ehrlichia* IgG, positive Rickettsia IgG and alpha-gal IgE > 0.1 IU/mL with symptomatic rKOA. The score count outcomes of severity PAS for hands, hips, and knees were each modeled using Zero-inflated negative binomial distribution to produce adjusted mean ratios (MR) and 95% CI to quantify the association of positive Ehrlichia IgG, positive Rickettsia IgG and Alpha-gal IgE > 0.1 IU/mL with PAS joint severity. The log of the mean PAS severity count is being modeled and the MR can be interpreted as how much worse the joint PAS severity is on average between two groups, for example how much worse the hand PAS severity is on average for participants with positive Ehrlichia IgG compared to participants negative. All of these sets of multivariable models were repeated replacing the dichotomous covariable Alpha-gal IgE > 0.1 IU/mL with a four-level variable: Alpha-gal IgE=0 IU/mL (reference), 0 IU/mL<Alpha-gal IgE≤0.1 IU/mL, 0.1 IU/mL<Alpha-gal IgE≤0.35 IU/mL, and 0.35 IU/mL<Alpha-gal IgE.

Multivariable models were used to assess the independent association of seroprevalence with the KOOS subscale scores. These models were adjusted for age, sex, race, BMI, education, smoking, alcohol use, Charlson comorbidity index. The score outcomes KOOS subscale were each modeled using Zero-inflated negative binomial distribution to produce adjusted MR and 95% CI to quantify the association of positive Ehrlichia IgG, positive Rickettsia IgG and Alpha-gal IgE > 0.1 IU/mL with KOOS subscale severity. The log of the mean KOOS subscale score is being modeled and the MR can be interpreted as how much worse the KOOS subscale score is on average between two groups, for example how much worse the KOOS Pain subscale score is on average for participants with positive Ehrlichia IgG compared to participants negative. To account for excess number of scores equal to 100, KOOS subscale scores were reversed as 100 minus score so the higher the score the worse the knee assessment. This allowed the fit of a zero-inflated model that can account for the excess zeros (in this case, corresponding to excess 100 scores).

© 2024 Zychowski DL et al. *JAMA Network Open*

eFigure 1. Ehrlichia and SFGR testing flowsheet.

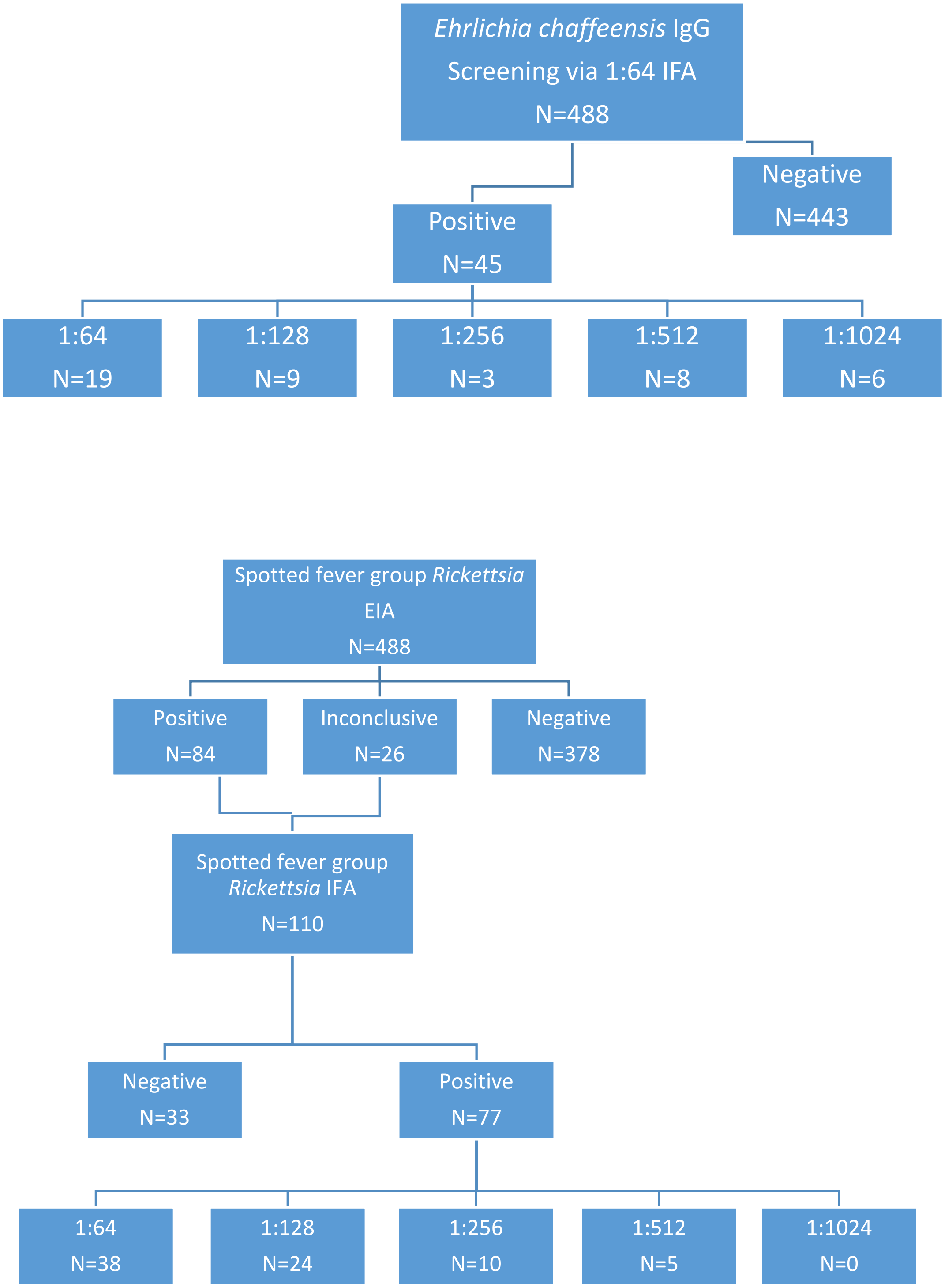

© 2024 Zychowski DL et al. *JAMA Network Open*

eFigure 2. Distribution of tick-borne serologic data. Distribution of positive tick-borne serologies among 488 participants.

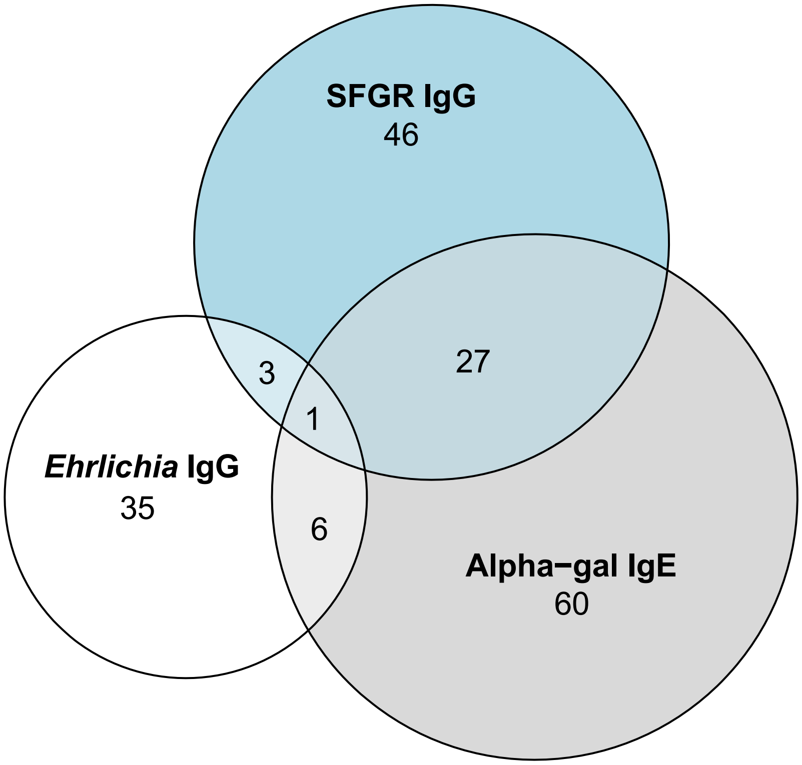

© 2024 Zychowski DL et al. *JAMA Network Open*

eTable 1. Alpha-gal and Total IgE levels. Distribution of alpha-gal IgE levels among 488 participants.

Characteristic		No. (%)<br>N=488
Alpha-gal IgE IU/mL		
	0 = Alpha-gal IgE	291 (59.6)
	0 < Alpha-gal IgE <0.1	98 (20.1)
	0.1 ≤ Alpha-gal IgE <0.35	34 (7.0)
	0.35 ≤ Alpha-gal IgE <2.0	44 (9.0)
	2.0 ≤ Alpha-gal IgE	21 (4.3)
Total IgE IU/mL, median (IQR)		40.8 (15.2-116)

Abbreviations: Alpha-gal, galactose-alpha-1,3-galactose; IgE, Immunoglobulin E

© 2024 Zychowski DL et al. *JAMA Network Open*

eTable 2. Univariate analyses

		Positive SFGR IgG		Positive *Ehrlichia* IgG		Alpha-gal IgE >0.1 IU/ml	
**Variable[a]**	**Modifier**	**Unadjusted OR**	**P value**	**Unadjusted OR**	**P value**	**Unadjusted OR**	**P value**
		% (95% CI)		*% (95% CI)*		*% (95% CI)*	
Age							
	1 year older	1.00 (0.97-1.04)	0.80	0.98 (0.94-1.02)	0.38	1.02 (0.99-1.05)	0.31
Sex							
	Male	3.28 (1.99-5.41)	<0.0001	0.69 (0.34-1.41)	0.31	3.25 (2.05-5.17)	<0.0001
	Female	Reference		Reference		Reference	
Race							
	Black	0.73 (0.42-1.25)	0.25	1.89 (1.02-3.52)	0.04	0.49 (0.28-0.83)	<0.01
	White	Reference		Reference		Reference	
Education							
	Less than HS education	0.69 (0.30-1.57)	0.37	1.70 (0.95-3.87)	0.20	1.21 (0.62-2.34)	0.58
	Greater than HS education	Reference		Reference		Reference	
BMI group							
	BMI (1 kg/m^2 higher)	0.99 (0.96-1.03)	0.67	0.99 (0.95-1.04)	0.78	0.98 (0.95-1.01)	0.23
Marital status							
	Married	2.20 (0.90-5.38)	0.08	0.83 (0.30-2.33)	0.73	1.59 (0.74-3.41)	0.24
	Never Married	0.82 (0.15-4.37)	0.81	2.20 (0.54-9.02)	0.27	0.81 (0.20-3.29)	0.77
	Separated	1.80 (0.18-18.08)	0.62	5.51 (0.80-37.86)	0.08	1.13 (0.12-10.87)	0.91
	Widowed	1.20 (0.44-3.25)	0.73	1.46 (0.51-4.22)	0.48	1.18 (0.51-2.74)	0.71
	Divorced	Reference		Reference		Reference	
Alcohol use							

© 2024 Zychowski DL et al. *JAMA Network Open*

	Current alcohol drinker	1.23 (0.67-2.26)	0.50	0.82 (0.35-1.90)	0.65	1.76 (1.03-3.0)	0.04
	Non drinker	Reference		Reference		Reference	
Smoking status							
	Current smoker	1.49 (0.59-3.82)	0.40	0.75 (0.17-3.25)	0.70	3.46 (1.58-7.59)	<0.01
	Non smoker	Reference		Reference		Reference	
Employment status							
	Retired	0.61 (0.33-1.15)	0.13	0.49 (0.23-1.03)	0.06	1.02 (0.54-1.93)	0.95
	Disabled	0.69 (0.23-2.07)	0.50	0.60 (0.16-2.34)	0.46	0.81 (0.26-2.49)	0.71
	Other	0.40 (0.05-3.36)	0.40	0.63 (0.07-5.45)	0.67	1.05 (0.20-5.52)	0.95
	Employed	Reference		Reference		Reference	
Family income							
	Annual family income < $45,000	0.64 (0.38-1.07)	0.09	1.82 (0.88-3.75)	0.11	0.90 (0.55-1.49)	0.69
	Annual family income > $45,000	Reference		Reference		Reference	
Charlson comorbidity index							
	3+	1.02 (0.50-2.06)	0.97	0.76 (0.27-2.16)	0.61	0.88 (0.44-1.77)	0.72
	2	0.35 (0.12-1.03)	0.06	2.39 (1.05-5.47)	0.04	1.28 (0.64-2.56)	0.49
	1	1.19 (0.68-2.09)	0.54	1.08 (0.50-2.32)	0.84	1.18 (0.69-2.01)	0.56
	0	Reference		Reference		Reference	
Age-adjusted charlson comorbidity index							

© 2024 Zychowski DL et al. *JAMA Network Open*

	3+<br>2<br>1	1.17 (0.14-9.83)<br>0.92 (0.10-8.49)<br>Reference	0.89<br>0.94	0.55 (0.06-4.69)<br>0.92 (0.10-8.49)<br>Reference	0.58<br>0.94	1.50 (0.18-12.58)<br>1.14 (0.13-10.36)<br>Reference	0.71<br>0.91
Blood Pressure							
	Elevated Blood Pressure[b]<br>Normal Blood Pressure	1.10 (0.66-1.83)<br>Reference	0.71	0.97 (0.51-1.87)<br>Reference	0.93	1.21 (0.76-1.93)<br>Reference	0.42
Diabetes							
	Diabetic<br>Non diabetic	0.85 (0.50-1.45)<br>Reference	0.55	1.36 (0.72-2.57)<br>Reference	0.34	1.05 (0.64-1.70)<br>Reference	0.86
CKD status							
	CKD<br>No CKD	0.73 (0.16-3.29)<br>Reference	0.69	1.39 (0.31-6.30)<br>Reference	0.67	N/A	0.97
Have you had an attached tick bite in the last 5 years?							
	Yes<br>No	2.44 (1.39-4.26)<br>Reference	<0.01	0.57 (0.22-1.50)<br>Reference	0.26	4.46 (2.67-7.46)<br>Reference	<0.0001
For those with an attached tick bite in the last 5 years, was there redness, itching or inflammation at the site of the bite? (N=84)							
	Yes<br>No	1.28 (0.44-3.76)<br>Reference	0.65	1.75 (0.19-16.45)<br>Reference	0.63	1.50 (0.57-3.93)<br>Reference	0.41

© 2024 Zychowski DL et al. *JAMA Network Open*

For those with an attached tick bite in the last 5 years who experienced redness, itching or inflammation at the site of the bite, did the reaction last less than a week? (N=59)							
	Yes<br>No	0.98 (0.30-3.19)<br>Reference	0.98	1.90 (0.25-14.53)<br>Reference	0.54	1.51 (0.52-4.42)<br>Reference	0.45
Do you think you have a food allergy?							
	Yes<br>No	0.70 (0.29-1.71)<br>Reference	0.44	1.10 (0.41-2.94)<br>Reference	0.84	1.37 (0.68-2.73)<br>Reference	0.38
Are there any foods you avoid because of a physical reaction?							
	Yes<br>No	0.55 (0.24-1.25)<br>Reference	0.16	0.91 (0.37-2.23)<br>Reference	0.83	1.05 (0.56-1.99)<br>Reference	0.87
Do you break out in hives sometimes?							
	Yes<br>No	0.26 (0.06-1.11)<br>Reference	0.07	2.77 (1.19-6.45)<br>Reference	0.02	1.24 (0.57-2.69)<br>Reference	0.59
Do you have a reaction to tick bites?							

© 2024 Zychowski DL et al. *JAMA Network Open*

	yes<br>no	2.93 (0.68-12.65)<br>Reference		N/A	0.98	0.96 (0.19-4.88)<br>Reference	0.96
Do you have a reaction to insect bites or spider bites?							
	yes<br>no	0.48 (0.17-1.39)<br>Reference	0.17	0.72 (0.21-2.43)<br>Reference	0.59	1.01 (0.47-2.19)<br>Reference	0.97
Do you have a reaction to stings?							
	yes<br>no	0.91 (0.50-1.67)<br>Reference	0.77	1.46 (0.73-2.91)<br>Reference	0.28	0.85 (0.49-1.50)<br>Reference	0.58
Do you eat red meat (this includes beef, pork, venison, lamb)?							
	Yes<br>No	1.27 (0.37-4.37)<br>Reference	0.71	1.07 (0.24-4.73)<br>Reference	0.93	1.14 (0.38-3.44)<br>Reference	0.81
Have you been diagnosed with alpha-gal allergy to red meat?							
	Yes<br>No	1.07 (0.23-4.97)<br>Reference	0.93	N/A		0.83 (0.18-3.87)<br>Reference	0.82
Have you had hand PAS in the past year?							
	Yes<br>No	1.11 (0.67-1.84)<br>Reference	0.70	0.52 (0.25-1.07)<br>Reference	0.07	0.92 (0.57-1.49)<br>Reference	0.74

© 2024 Zychowski DL et al. *JAMA Network Open*

Have you had hip PAS in the past year?							
	Yes<br>No	0.70 (0.40-1.22)<br>Reference	0.21	1.14 (0.59-2.19)<br>Reference	0.69	1.07 (0.66-1.74)<br>Reference	0.78
Have you had knee PAS in the past year?							
	Yes<br>No	1.42 (0.87-2.32)<br>Reference	0.16	0.50 (0.25-0.99)<br>Reference	<0.05	1.04 (0.66-1.64)<br>Reference	0.88
Alpha-gal IgE							
	>0.1 IU/mL<br>≤0.1 IU/mL	2.99 (1.75-5.09)	<0.0001	0.75 (0.33-1.75)	0.51	N/A	

Abbreviations: BMI, body mass index; HS, high school; CKD, chronic kidney disease.
[a]: Unless otherwise indicated, data are expressed as No. (%) of participants. Percentages have been rounded and may not total to 100.
[b]: Elevated blood pressure defined by systolic blood pressure ≥140mm Hg or diastolic blood pressure ≥90mm Hg.

© 2024 Zychowski DL et al. *JAMA Network Open*

eTable 3. Multivariable models for KOOS

Covariables	KOOS Symptoms	KOOS Stiffness	KOOS Pain	KOOS Physical Function Short	KOOS Quality of Life
	MR (95% CI)	MR (95% CI)	MR (95% CI)	MR (95% CI)	MR (95% CI)
Positive *Ehrlichia* IgG	0.95 (0.73, 1.24)	0.71 (0.58, 0.87)	0.96 (0.70, 1.31)	1.01 (0.77, 1.33)	0.99 (0.82, 1.19)
Positive SFGR IgG	0.95 (0.78, 1.15)	0.96 (0.82, 1.11)	1.00 (0.80, 1.25)	1.06 (0.87, 1.29)	1.07 (0.93, 1.23)
Alpha-gal IgE > 0.1 IU/mL	1.05 (0.87, 1.27)	1.06 (0.91, 1.23)	1.31 (1.05, 1.62)	1.21 (1.01, 1.45)	1.18 (1.03, 1.35)

Abbreviations: KOOS, Knee injury and Osteoarthritis Outcome Scores; MR, Mean ratio; CI, confidence interval.

© 2024 Zychowski DL et al. *JAMA Network Open*

eTable 4. Multivariable models for symptomatic radiographic osteoarthritis as well as Hands, Hips and Knees PAS

Covariables	Hands PAS severity	Hips PAS severity	Knees PAS severity	Symptomatic rKOA
	MR (95% CI)	MR (95% CI)	MR (95% CI)	OR (95% CI)
Positive *Ehrlichia* IgG	0.88 (0.65, 1.17)	0.84 (0.66, 1.07)	0.98 (0.73, 1.32)	0.69 (0.30, 1.60)
Positive SFGR IgG	1.14 (0.95, 1.37)	0.89 (0.72, 1.11)	1.05 (0.88, 1.26)	1.65 (0.92, 2.98)
Alpha-gal IgE > 0.1 IU/mL	1.19 (1.00, 1.42)	1.18 (0.98, 1.43)	1.30 (1.09, 1.56)	0.78 (0.43, 1.43)
Positive *Ehrlichia* IgG (vs negative)				
Titer = 1:64	0.87 (0.58, 1.31)	0.75 (0.55, 1.03)	1.07 (0.69, 1.64)	not estimable
Titer 1:128 – 1:1024	0.88 (0.58, 1.31)	1.01 (0.71, 1.45)	0.89 (0.61, 1.30)	not estimable
Positive SFGR IgG (vs negative)				
Titer = 1:64	1.24 (0.98, 1.56)	0.85 (0.65, 1.11)	1.19 (0.95, 1.49)	2.69 (1.19, 6.05)
Titer 1:128 – 1:512	1.03 (0.79, 1.33)	0.97 (0.69, 1.36)	0.89 (0.68, 1.16)	1.10 (0.50, 2.44)
Alpha-gal (vs 0 IU/mL)				
>0 – 0.1 IU/mL	1.02 (0.86, 1.22)	0.92 (0.76, 1.12)	1.14 (0.95, 1.37)	0.94 (0.53, 1.65)
>0.1 – 0.35 IU/mL	1.26 (0.99, 1.60)	1.24 (0.95, 1.62)	1.19 (0.90, 1.57)	0.76 (0.29, 2.01)
>0.35 IU/mL	1.18 (0.92, 1.52)	1.09 (0.85, 1.38)	1.49 (1.19, 1.85)	0.81 (0.38, 1.69)

Abbreviations: MR, mean ratio; CI, confidence interval; PAS, pain, aching, stiffness; rKOA, radiographic knee osteoarthritis.

Models adjusted by age, BMI, sex, race, education, smoking status, alcohol use, Charlson comorbidity index, and other listed covariates. The first set of models, by outcome, include the covariables listed in the first three rows; the remaining models include the covariables in the latter rows.

© 2024 Zychowski DL et al. *JAMA Network Open*
