# Supplementary material for: Tick-Borne Disease Infections and Chronic Musculoskeletal Pain
Source: JAMA Netw Open. 2024 Jan 11;7(1):e2351418. doi: 10.1001/jamanetworkopen.2023.51418 (PMC10784854; doi:10.1001/jamanetworkopen.2023.51418)
Supplement: Supplement 2. — Data Sharing Statement [file jamanetwopen-e2351418-s002.pdf]

Data Sharing Statement

Zychowski. Tick-Borne Disease Infections and Chronic Musculoskeletal Pain. *JAMA Netw Open*. Published January 11, 2024. doi:10.1001/jamanetworkopen.2023.51418

Data
Data available: Yes
Data types: Deidentified participant data
How to access data: Deidentified individual data that supports the results will be shared beginning 9 to 36 months following publication provided the investigator who proposes to use the data has approval from an Institutional Review Board (IRB), Independent Ethics Committee (IEC), or Research Ethics Board (REB), as applicable, and executes a data use/sharing agreement with UNC.
When available: beginning date: 05-28-2024, end date: 08-28-2026

Supporting Documents
Document types: None

Additional Information
Who can access the data: Researchers whose proposed use of the data has been approved from an Institutional Review Board (IRB), Independent Ethics Committee (IEC), or Research Ethics Board (REB), as applicable, and executes a data use/sharing agreement with UNC.
Types of analyses: Data will be available for research purposes.
Mechanisms of data availability: Researchers whose proposed use of the data has been approved from an Institutional Review Board (IRB), Independent Ethics Committee (IEC), or Research Ethics Board (REB), as applicable, and executes a data use/sharing agreement with UNC.
